# Methylation profiling of normal individuals reveals mosaic promoter methylation of cancer-associated genes

**DOI:** 10.18632/oncotarget.480

**Published:** 2012-05-08

**Authors:** Lasse Sommer Kristensen, Michael Raynor, Ida Candiloro, Alexander Dobrovic

**Affiliations:** ^1^ Molecular Pathology Research and Development Laboratory, Department of Pathology, Peter MacCallum Cancer Centre, Melbourne, Australia; ^2^ Department of Biomedicine, Aarhus University, Denmark; ^3^ Department of Haematology/Oncology, The Queen Elizabeth Hospital, Adelaide, South Australia, Australia; ^4^ Department of Pathology, The University of Melbourne, Parkville, Australia; ^5^ Sir Peter MacCallum Department of Oncology, The University of Melbourne, Parkville, Australia

**Keywords:** DNA methylation, epigenetics, biomarkers, high-resolution melting (HRM), methylation-specific PCR (MSP), constitutional methylation

## Abstract

Epigenetic silencing by promoter methylation of genes associated with cancer initiation and progression is a hallmark of tumour cells. As a consequence, testing for DNA methylation biomarkers in plasma or other body fluids shows great promise for detection of malignancies at early stages and/or for monitoring response to treatment. However, DNA from normal leukocytes may contribute to the DNA in plasma and will affect biomarker specificity if there is any methylation in the leukocytes. DNA from 48 samples of normal peripheral blood mononuclear cells was evaluated for the presence of methylation of a panel of DNA methylation biomarkers that have been implicated in cancer. SMART-MSP, a methylation specific PCR (MSP) methodology based on real time PCR amplification, high-resolution melting and strategic primer design, enabled quantitative detection of low levels of methylated DNA. Methylation was observed in all tested mononuclear cell DNA samples for the *CDH1* and *HIC1* promoters and in the majority of DNA samples for the *TWIST1* and *DAPK1* promoters. *APC* and *RARB* promoter methylation, at a lower average level, was also detected in a substantial proportion of the DNA samples. We found no *BRCA1*, *CDKN2A*, *GSTP1* and *RASSF1A* promoter methylation in this sample set. Several individuals had higher levels of methylation at several loci suggestive of a methylator phenotype. In conclusion, methylation of many potential DNA methylation biomarkers can be detected in normal peripheral blood mononuclear cells, and is likely to affect their specificity for detecting low level disease. However, we found no evidence of promoter methylation for other genes indicating that panels of analytically sensitive and specific methylation biomarkers in body fluids can be obtained.

## INTRODUCTION

Recent research has led to the understanding that, despite the importance of genetic change, epigenetic mechanisms are perhaps the predominant drivers of cancer [1-3]. In particular, inactivation of genes by *de novo* promoter methylation accompanied by global hypomethylation is the most common DNA lesion of cancer cells [2, 3].

The CpG dinucleotide is the principal unit of methylation in humans as its palindromic pairing with a CpG dinucleotide on the other strand enables the semiconservative replication of methylation by DNA methyltransferase 1. Regions of high CpG density are referred to as CpG islands. CpG islands are often found spanning the promoter and first exon of genes, and these promoter region CpG islands are normally unmethylated. DNA methylation of promoter regions is strongly associated with transcriptional silencing of the downstream gene. This may in part be explained by the binding of proteins containing methyl binding domains which then initiate the formation of complexes that repress transcription [4].

*De novo* methylation of CpG islands is often an early event in cancer [5]. Thus aberrant promoter methylation of a number of genes shows great promise as biomarkers for early cancer detection [5-7]. Ideally methylation biomarkers should be detectable in readily accessible body fluids or tissues. However, the majority of DNA methylation biomarkers lack the sensitivity and specificity required for a diagnostic test [7,8].

Plasma and serum are the most studied body fluids with regard to DNA methylation biomarkers. Very sensitive and quantitative methods are needed for the detection of these biomarkers. However, tumour-derived DNA in body fluids may often be significantly contaminated with DNA from normal cells especially when the tumour is small. Though the source of the normal DNA is unclear, it is likely that DNA from normal leukocytes may affect biomarker specificity if methylated.Thus, very sensitive and quantitative methods may be needed for the detection of these biomarkers in body fluids.

Biomarker sensitivity and specificity is also influenced by the choice of region for analysis and the choice of method for methylation analysis. Many different methods for the detection of DNA methylation are available [9, 10]. The most sensitive methods utilise methylation-specific PCR (MSP) [11] in which amplification is determined by the methylation of the region under the primers. Conventional MSP is widely used due to its simplicity. However, it is non-quantitative and is also prone to false positive results caused by incomplete bisulphite conversion or false priming events [5, 9, 12-14]

Thus, when it is necessary to detect rare methylated sequences, MSP may be unable to distinguish low-level methylation from low-level incomplete conversion or false priming. MethyLight, a variant of MSP using fluorescent TaqMan probes is more specific [15] as the use of the probe makes it quantitative and enables increased specificity.

We recently developed a highly sensitive probe-free method called Sensitive Melting Analysis after Real Time (SMART)-MSP [12], which can provide quantitative data, works well with DNA derived from formalin fixed paraffin embedded (FFPE) tissues [16], and is capable of identifying many false positives resulting from false priming or incomplete conversion of unmethylated cytosines during the bisulphite conversion of the DNA template. This is possible due to the use of high-resolution melting analysis [17], which utilises intercalating dyes that can be used at saturating conditions without inhibiting the PCR [18].

In this contribution, we have evaluated the methylation levels in normal peripheral blood mononuclear cells for a panel of genes (*APC*, *BRCA1*, *CDH1*, *CDKN2A*, *DAPK1*, *GSTP1*, *HIC1*, *RARB*, *RASSF1A* and *TWIST1*). These genes have been implicated as actual or potential tumour suppressor genes and are methylated in various cancers. These genes have potential as DNA methylation biomarkers for both early detection and post-therapeutic monitoring. By using SMART-MSP we were able to distinguish artefactual results from true positive results, and to obtain quantitative data for the studied panel of biomarkers. Thus, we were able to show that some are normally methylated at low levels in peripheral blood mononuclear cells, whereas methylation was undetectable in others.

## RESULTS

### Melting profiles of the SMART-MSP assays

The melting profiles of true positives for each assay were obtained by amplifying methylated standards. The melting temperature (T_m_) was 78.3°C for *APC*, 76.5°C for *BRCA1*, 81.9°C for *CDH1*, 75.8°C for *CDKN2A*, 79.5°C for *DAPK1*, 80°C for *GSTP1*, 79.8°C for *HIC1*, 81.5°C for *RARB*, 78.2°C for *RASSF1A* and 79.8°C for *TWIST1*. Only bands of the expected size were observed after electrophoresis (data not shown).

### The sensitivity and quantitative accuracy of the SMART-MSP assays

The sensitivity of the SMART-MSP assays was tested using a standard dilution series of methylated DNA into unmethylated DNA. The *CDKN2A* and the *RASSF1A* assays were sensitive to 0.1% methylated template with high reproducibility. The *APC*, *BRCA1*, *CDH1*, *DAPK1*, *GSTP1*, *HIC1*, *RARB* and *TWIST1* assays were sensitive to 0.05% methylated template (data not shown).

All standards contained approximately equal amounts of template suitable for PCR after bisulphite conversion, as evident from the similar C_T_ values obtained in the *COL2A1* control assay. We used the Rotorgene software to obtain a standard curve for the dilution series for calculation of the correlation coefficient (r^2^) and PCR efficiency (*E*) for each assay. The correlation coefficients (*APC*: r^2^ = 0.987, *BRCA1*: r^2^ = 0.985, *CDH1*: r^2^ = 0.992, *CDKN2A*: r^2^ = 0.998, *DAPK1*: r^2^ = 0.995, *GSTP1*: r^2^ = 0.985, *HIC1*: r^2^ = 0.995, *RARB*: r^2^ = 0.982, *RASSF1A*: r^2^ = 0.978, *TWIST1*: r^2^ = 0.995) indicated a strong linear relationship between C_T_ values and given concentrations for all assays. The PCR efficiency of most assays (*APC*: *E* = 1.89, *CDH1*: *E* = 1.88, *CDKN2A* E = 1.96, *DAPK1*: *E* = 1.86, *GSTP1*: *E* = 1.94, *HIC1*: *E* = 1.87, *RARB*: *E* = 1.92, *TWIST1*: *E* = 1.88) was approximately the same as the control assay (*COL2A1*: *E* =1.90). The PCR efficiencies of the *BRCA1* (*E* = 1.78) and *RASSF1A* (*E* = 1.98) assays were more different from the control assay, but as none of the samples were positive for these assays, quantification was not necessary.

### Promoter methylation levels in the samples

The *COL2A1* control assay was used to normalise for DNA input after bisulphite conversion in the real-time PCR quantification [12]. The samples generally amplified simultaneously and relatively early with the *COL2A1* assay. One sample (#45) that amplified late for the *COL2A1* assay, (indicating a very low number of target bisulphite modified templates) was omitted from the analysis.

Quantitative data for all samples for each assay is shown in Table 2. None of the samples were positive for *BRCA1*, *CDKN2A*, *GSTP1* or *RASSF1A* methylation in the regions analysed. The regions analysed for the other genes were detectably methylated in some or all of the individuals tested: *HIC1*: 100%, *CDH1*: 100%, *TWIST1*: 72%, *DAPK1*: 51%, *RARB*: 32%, *APC*: 23%.

**Table 1:** Primer sequences, annealing temperatures, and amplicon information for the SMART-MSP assays

Gene	Primer sequences (CpG sites in bold and converted Cs as capital Ts or As)	Annealing temperature(°C)	Size(bp)	Non-CpG Cs between primers	Spanned region (UCSC Genome Browser, March 2006: NCBI36/hg18)
***APC***	F-tT**cg**Ttggatg**cg**gaTTaggg**c** R-ccaat**cg**A**cg**AActcc**cg**a**cg**	68	55	7	112101367- 112101421 Chr. 5
***BRCA1***	F-tgTttag**cg**gtagTTTTttggtttT**c** R-ttcc**cgcg**cttttc**cg**	65	49	1	38530948- 38530996 Chr. 17
***CDH1***	F-gtggg**cg**ggT**cg**tTagTtT**c** R-AccacaAccaatcaAcaA**cgcg**A	65/68	58	3	67328555-67328612 Chr. 16
***CDKN2A***	F-gTaTTtTTtT**cg**agTaTt**cg**TtTa**cg**g**c** R-caaatcctctAAaAAAac**cgcg**A	64	72	6	21964971- 21965042 Chr. 9
***DAPK1***	F-aggaTagT**cg**gaT**cg**agTTaa**cg**T**c** R-ttAc**cg**aAtcccctc**cgcg**A	67	61	4	89302618- 89302678 Chr. 9
***GSTP1***	F-g**cg**aTtT**cg**gggaTtTTaggg**c** R-tAcaccc**cg**AA**cg**t**cg**Ac**cg**	67	51	5	67107679- 67107729 Chr. 11
***HIC1***	F-TTagg**cg**gTTaggg**cg**T**cg**Ta**c** R-ctA**cg**AAAacacacac**cg**Ac**cg**A	66	54	4	1906662-1906714 Chr. 17
***RARB***	F-atgT**cg**agaa**cgcg**ag**cg**atT**c** R-gttc**cg**Aatcctaccc**cg**a**cg**A	71	64	3	25444860-25444923 Chr. 3
***RASSF1A***	F-**cg**TT**cg**gTT**cgcg**TttgTtag**c** R-tAAcc**cg**AttAAAcc**cg**tActt**cg**	68	58	5	50353240- 50353297 Chr. 3
***TWIST1***	F-**cgcg**gTTaggaTagtTtTTtT**cg**aT**c** R-aA**cg**cccc**cg**aaccctaA**cg**	68	59	4	19124102-19124160 Chr. 7
***COL2A1***	F-gTaatgTTaggagTaTTTtgtgggTa R-ctaccccaAAaAaAcccaAtcctA	65	86	1	46667210- 46667295 Chr. 12

It should be noted that optimal annealing temperature may vary according to the PCR machine used and the other parameters of the PCR reaction.

**Table 2:** Summary of the DNA methylation levels estimated by SMART-MSP for each sample ordered according to age

Sample (Age/Sex)	APC	CDH1	DAPK1	HIC1	RARB	TWIST1
**1 (84/M)**	0.09%	0.52%	0.16%	2.72%	0%	0.05%
**2 (84/M)**	0%	1.68%	0.14%	2.21%	0.06%	0.21%
**3 (83/F)**	0.06%	1.18%	0.17%	1.46%	0.13%	0.32%
**4 (82/M)**	False positive	0.64%	0.06%	0.96%	0%	0.17%
**5 (82/F)**	0.24%	1.18%	0.14%	3.13%	0.06%	0.39%
**6 (80/F)**	0%	3.85%	0.32%	7.69%	0.30%	0.48%
**7 (77/M)**	0%	3.35%	0.11%	8.25%	0.28%	0.16%
**8 (76/M)**	0%	0.78%	0%	1.46%	0%	0.17%
**9 (75/F)**	0%	1.68%	0.18%	12.50%	0%	0.09%
**10 (74/M)**	0.21%	4.42%	0.39%	12.50%	0.48%	2.06%
**11 (71/M)**	0.52%	5.83%	0.17%	20.31%	0.45%	7.18%
**12 (71/F)**	0%	1.79%	0.07%	1.92%	0%	0.07%
**13 (71/F)**	0%	2.21%	0%	4.74%	0%	0%
**14 (69/M)**	0%	1.27%	0%	1.92%	0%	0.05%
**15 (68/M)**	0%	1.27%	0%	14.36%	0.21%	0.42%
**16 (68/M)**	0,09%	0.73%	0%	3.13%	0.10%	0.15%
**17 (66/F)**	False positive	1.11%	False positive	2.06%	0%	0.16%
**18 (66/M)**	0%	1.18%	0%	5.83%	0.07%	0.24%
**19 (61/F)**	0%	1.56%	0.09%	1.79%	False positive	0.05%
**20 (61/F)**	0.08%	0.96%	0%	2.92%	0.05%	0.36%
**21 (60/M)**	0.10%	0,59%	0.05%	2.37%	0.13%	0.05%
**22 (58/M)**	False positive	0.84%	0.06%	1.46%	0%	0%
**23 (56/M)**	0%	0.45%	0%	0.73%	0%	0.26%
**24 (55/M)**	0%	0.84%	0.06%	3.35%	0%	0.21%
**25 (54/F)**	0%	1.03%	0.05%	1.27%	0,06%	0.18%
**26 (52/F)**	0%	0.36%	0%	1.67%	False positive	0%
**27 (50/F)**	False positive	0.48%	0%	1.36%	0%	0.06%
**28 (50/M)**	0%	1.27%	0.21%	12.50%	0%	0%
**29 (49/F)**	0%	0.73%	0%	1.46%	0%	0.12%
**30 (48/M)**	0%	0.28%	0%	4.12%	0%	0%
**31 (45/F)**	0.26%	2.06%	0.06%	1.67%	0%	0.10%
**32 (44/M)**	0%	1.92%	0.13%	3.35%	0.16%	0.10%
**33 (42/F)**	0.06%	1.03%	0.18%	1.10%	0%	0.15%
**34 (39/F)**	0%	0.36%	0%	1.92%	0%	0.16%
**35 (37/M)**	0%	0.48%	0%	1.36%	0%	0%
**36 (33/M)**	0%	1.56%	0%	2.21%	0%	0.11%
**37 (33/F)**	0%	0.52%	0%	1.27%	0%	0%
**38 (33/M)**	False positive	0.89%	0%	1.56%	0%	0.11%
**39 (29/F)**	0%	0.28%	0%	1.46%	0%	0%
**40 (27/M)**	0%	0.52%	0%	2.21%	0%	0%
**41 (26/F)**	0.09%	1.18%	0.06%	1.67%	0%	0.06%
**42 (26/M)**	0%	0.45%	0%	0.78%	0%	0.05%
**43 (26/F)**	0%	0.96%	0.08%	1.18%	False positive	False positive
**44 (25/M)**	0%	1.68%	0.13%	2.06%	0.13%	0.10%
**45 (23/M)**	No data	No data	No data	No data	No data	No data
**46 (23/F)**	0%	0.36%	0%	0.78%	0%	0%
**47 (23/M)**	0%	0.63%	0.06%	1.79%	0%	False positive
**48 (?/?)**	False positive	0,24%	0%	1,18%	False positive	0%

Some regions tended to be methylated at higher levels i.e. in a higher proportion of cells than others. The estimated methylation levels were highest for the *HIC1* and *CDH1* assays. *TWIST1, APC*, *DAPK1* and *RARB* positive samples were methylated at lower levels. The mean methylation level of positive samples for each gene was: *HIC1*: 3.61%, *CDH1*: 1.26%, *TWIST1*: 0.43%, *RARB*: 0.18%, *APC*: 0.18%, *DAPK1*: 0.13%. The overall level of methylation observed in the positive samples was higher for the assays that were positive in a greater proportion of samples. Thus, it is possible that each gene’s methylation may represent a continuous variable with the more highly methylated genes having the whole range above the threshold of detection.

**Figure 1:**
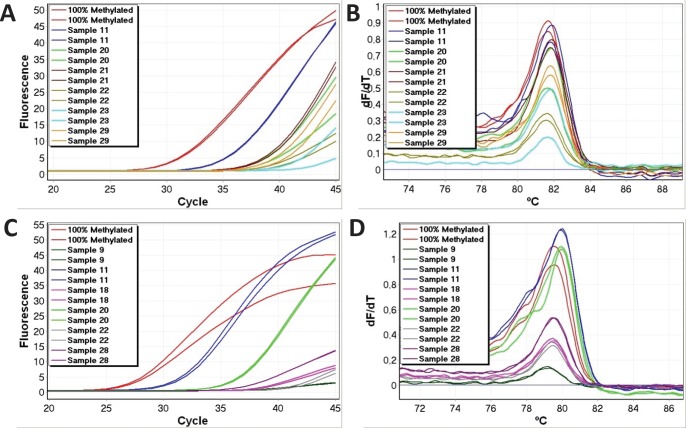
Representative methylation positive samples for CDH1 and TWIST1 Good reproducibility and non-shifted melting profiles were observed, and were used as criteria for identification of true positive results for all genes. A. Real time amplification data (*CDH1*). B. High-resolution melting profiles (*CDH1*). C. Real time amplification data (*TWIST1*). D. High-resolution melting profiles (*TWIST1*).

The range of methylation levels varied the most for the *HIC1* assay (many samples were far from the mean). The methylation levels also varied considerably for the *CDH1* and *TWIST1* assays. Real-time amplification data and melting profiles for representative samples are shown for *CDH1* and *TWIST1* (Figure 1).

A tendency towards increasing methylation levels with increasing age was observed for most of the genes. However, more samples would be needed to adequately address this question. Two samples (10 and 11) showed overall elevated methylation levels for most of the assayed genes. These samples were not only methylated at high levels but also had a large number of methylated genes. In contrast, samples 26, 27, 35, 37, 39, 42, 46, 47 and 48 overall showed low methylation levels and methylation was restricted to a few genes (Table 2).

The reproducibility was not as good for the replicates showing the lowest levels of DNA methylation as evident from figure 1A, and occasionally only one replicate amplified. This can be readily explained by the limiting amounts of methylated template at the lowest methylation levels. The quantitative data in Table 2 is calculated from the mean values of the replicates. In situations where only one of the two replicates amplified, the methylation level estimated from that replicate was halved.

### Identification of false positives caused by incomplete conversion

In the SMART-MSP assays used, only non-CpG cytosines are located between the primers. Thus, if these cytosines are not converted to uracil during the bisulphite modification, the amplicon will have a higher GC-content, melt later, and thus a right-shifting (due to a higher than expected melting temperature) of the melting profile will be observed. This, in combination with late amplification is a strong indicator of a false positive result [12].

**Figure 2:**
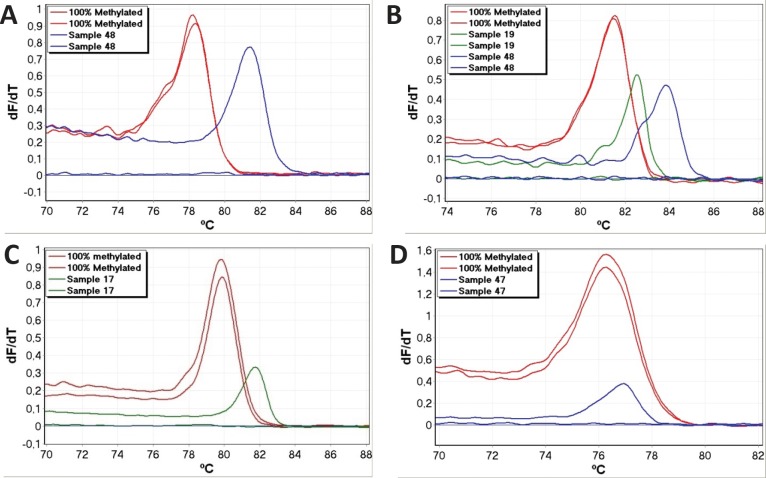
Examples of false positive results False positives caused by incomplete bisulphite conversion were identified as right-shifted melting profiles (relative to the 100% methylated standard). These were associated with late amplification and poor reproducibility. All shown samples would score as positives when using gel electrophoresis. A. The *APC* SMART-MSP assay. B. The *RARB* SMART-MSP assay. C. The *DAPK1* SMART-MSP assay. D. The *BRCA1* SMART MSP assay.

Occasionally, amplified samples showed markedly right-shifted melting profiles, and were scored as false positives (Table 2). Examples from the *APC*, *BRCA1*, *DAPK1* and *RARB* assays are shown in figure 2. Right-shifting was always associated with late amplification and poor reproducibility between replicates as would be expected for this type of false positive result [12]. This is because incompletely converted molecules are typically found at low levels. These samples would have been scored as positive results when using gel electrophoresis as in conventional MSP because gel electrophoresis sorts DNA molecules according to size, and does not evaluate the bases found in between the primers as the SMART-MSP assays do.

**Figure 3:**
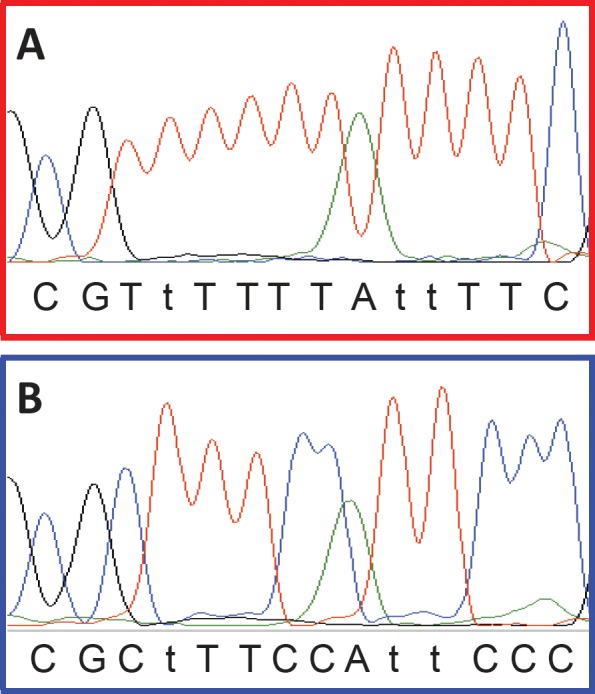
Sequencing confirms that right-shifting was due to incomplete conversion In the *APC* SMART-MSP assay seven non-CpG cytosines are found in between the primers (shown as upper case T’s when converted during the bisulphite modification). The last base of each primer and the region in between them are shown here. CpG sites are shown in bold. A. Sequencing results for the 100% methylated control. All non-CpG cytosines were converted during the bisulphite modification. B. Sequencing results for sample 48 from figure 2A. It is observed that five out of the seven non-CpG cytosines found in between the primers were not converted. This is causing the right-shift observed in figure 2A.

Samples were scored as a false positive result in situations where both replicates amplified and showed right-shifted melting and in situations where only one of the replicates amplified and showed a right-shifted melting profile while the other replicate did not amplify. Variant *APC* products were sequenced to confirm that right-shifting was due to incomplete conversion. An example is shown for sample 48 in figure 3 (also shown in figure 2A). It is observed that five out of the seven non-CpG cytosines found in between the *APC* primers were not converted, whereas all seven sites were converted in the positive control.

### Verification of CDH1 results by MethyLight

**Figure 4:**
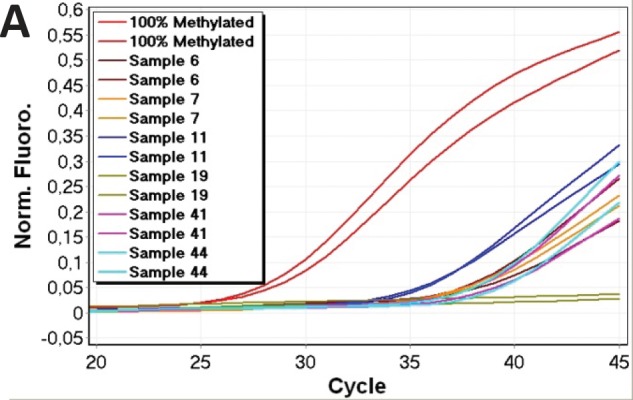
Verification of CDH1 results using MethyLight Normalised amplification data for six of the 14 samples analysed by MethyLight are shown. MethyLight results were consistent with the SMART-MSP results. However, sample 19 did not amplify in the MethyLight assay. This indicates that the two additional CpG positions analysed by the MethyLight assay are rarely methylated in this sample.

The *CDH1* MethyLight assay was quantitatively accurate in the range from 100% down to 0.1% methylated template. The correlation coefficient of the MethyLight assay was: r^2^ = 0.984, again indicating a strong linear relationship between C_T_ values and given concentrations. We tested a selection of the samples showing different levels of *CDH1* methylation (sample 5, 6, 7, 10, 11, 13, 14, 19, 20, 36, 41, 43, 44, 46) using MethyLight to confirm that these positive results were not a result of false priming. In the MethyLight assay, CpG sites are also present in the probe sequence which increases the specificity for methylated templates. All of the samples, except sample 19 which was negative, were shown to be methylated at similar but slightly lower levels compared to the data obtained by the *CDH1* SMART-MSP assay. MethyLight amplification data for six of the samples are shown in figure 4.

### CDH1 SMART-MSP using different annealing temperatures

If the observed methylation with the *CDH1* assay was due to a rare subpopulation of fully methylated cells, the samples would be estimated to be methylated at the same levels when using a higher annealing temperature. We tested a number of the samples showing different levels of *CDH1* methylation (sample 2, 3, 5, 10, 11, 12, 15, 18, 20, 37) using 68°C as the annealing temperature instead of 65°C. Again, we found high reproducibility and non-shifted melting profiles (data not shown). However, we found that all samples were methylated at similar but slightly lower levels. These results and the MethyLight results indicate that there is some heterogeneity in the methylation. As the temperature is increased, tolerance of the primers binding to an imperfectly matched template is decreased.

### Confirming that CDH1 is heterogeneously methylated by HRM

To further test whether the observed *CDH1* methylation is heterogeneous, we used the same primers as for the MethyLight assay, but with the dye SYTO-9 instead of the probe. This allows evaluation of the methylation status of the CpG sites between the primers including the two CpG sites covered by the MethyLight probe. If left-shifted melting profiles relative to the 100% methylated standard are observed this is an indication that the two CpG sites are heterogeneously methylated, due to a less GC-rich amplicon and/or heteroduplex formation [12].

**Figure 5:**
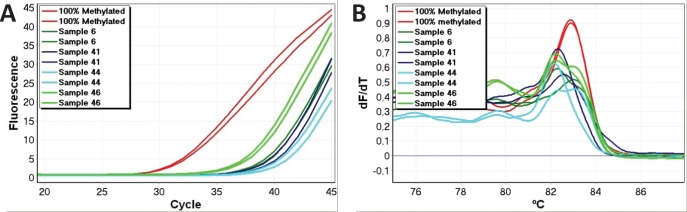
Assessment of the methylation status of the two CpG positions found in between the second set of CDH1 primers by HRM Left-shifted melting profiles relative to the 100% methylated standards were observed. This is an indication that the two CpG sites are not consistently methylated in all of the amplified molecules. A. Amplification data. B. High resolution melting profiles.

We tested a number of the samples (sample 6, 7, 11, 13, 19, 36, 41, 43, 44, 46) and found broader melting profiles extending to the left for some of them (Figure 5), and some displayed a twin peak. This was interpreted as the amplification of molecules having both CpG sites methylated as well as molecules having neither or one of the two CpG sites between the primers methylated.

## DISCUSSION

We found no evidence for methylation of the *CDKN2A*, *GSTP1, RASSF1A* and *BRCA1* promoter regions assayed in this set of peripheral blood mononuclear cell DNA samples. It is instructive that these biomarkers for which we found no methylation have been successfully used in a number of other studies. As examples, methylation of the *CDKN2A* promoter has been detected in sputum of smokers up to 3 years before they were diagnosed with cancer [19, 20], *GSTP1* has been used in prostate cancer detection [21], and *RASSF1A* can be detected in serum from gastric colorectal adenocarcinoma patients [22]. Women with detectable *BRCA1* methylation in the peripheral blood have an increased risk of early onset breast cancer [23].

However, we have shown that significant levels of mosaic promoter CpG island methylation are present in peripheral blood mononuclear cells derived from normal individuals for a number of other genes whose methylation is implicated in cancer. These results have several important implications.

Firstly, they indicate that the assays that show considerable background methylation will be insufficiently specific for use as DNA methylation biomarkers for peripheral blood–based early detection or monitoring. Similar considerations apply for monitoring of body fluids or tissues that have significant a haematopoietic component but these will require further analysis of background methylation in other contributing tissues. Thus, our results support the use of assays based on *BRCA1*, *CDKN2A*, *GSTP1* and *RASSF1A* methylation as specific biomarkers. However, assays based on *APC*, *CDH1*, *DAPK1*, *HIC1*, *RARB* and *TWIST1* methylation may prove to be less specific biomarkers, especially if non-quantitative methods are used.

One reservation about this study using peripheral blood mononuclear cells is that the neutrophils which comprise the major leukocyte component of blood were not analysed. Future studies will need to investigate blood fractions more intensively as well as investigating variations in other tissues.

Some of the genes with substantial background methylation in this study have been used for DNA methylation biomarkers. For example, *TWIST1* and *RARB* methylation was used to detect breast cancer cells in ductal lavages [24]. Whereas, the assays used were different from ours and may have been more specific under the reaction conditions used, it is clear that each assay for a methylation biomarker needs to be thoroughly tested against population controls and appropriate tissues as a critical step in its validation.

Among the panel of genes that we have found to be methylated, *CDH1* [25] and *DAPK1* [26] have previously been reported to be methylated in the blood in a high proportion of normal individuals. *CDH1* was reported to be heterogeneously methylated as also found in the present study. The use of less sensitive methods and/or the use of different blood fractions and a lack of sufficient normal controls may explain why the other genes for which we observed significant methylation have not previously been shown to be methylated in normal blood. Furthermore, positives obtained by conventional MSP may have been considered as false positive results. In this study, false positive results that are likely to be caused by incomplete conversion were readily identified. By contrast, identification of false positives is not possible using conventional MSP.

The use of a whole genome amplified (WGA) unmethylated control allowed us to determine the appropriate PCR conditions for minimising false priming events. However, even after optimisation the WGA control occasionally amplified. This was associated with a lack of reproducibility in the replicates, very late amplification, and right-shifted melting profiles. As no CpG sites were placed in between most pairs of SMART-MSP primers, this allowed us to conclude that the observed right-shifting is caused by incomplete conversion of some of the non-CpG cytosines found between the primers. This was verified by sequencing for the APC SMART-MSP assay.

Similar false positive results likely to be due to the amplification of very low-level incompletely converted templates were observed in the samples. When these false positive reactions were analysed by electrophoresis, a fragment of the same size as the 100% methylated positive control was observed. These samples would have scored as positive by conventional MSP, however, the number of non-CpG cytosines between the primers influences the likelihood of detecting these false positives. In our *BRCA1* samples, only one non-CpG cytosine is located between the primers, due to restrictions in assay design caused by the *BRCA1* pseudogene[23]. Amplification occurred in 3 samples and the melting profiles were right-shifted by 0.6-0.8°C. Given the size of the amplicon a shift in this range is expected for a single C to T change [27,28]. Thus, these samples were scored as false positive results. However, for the other assays with more non-CpG cytosines between the primers, the vast majority of samples we scored as false positive results showed much larger right-shifts (generally between 1°C to 4°C) indicating that several of the cytosines were not converted.

Small right-shifts below 0.6°C were observed for some samples (Figure 1B and D). This was a consequence of the bisulphite conversion procedure being done using columns for the standards and using plates for the samples. The precise kit used for bisulfite modification affects the melting results probably as a result of different salt concentrations (unpublished results). This correctable fault points out the importance of using the exact same kit for template production for all high resolution melting experiments.

We observed the highest false positive rates in the *APC* and *RARB* assays. This can be explained by the primers for these assays containing fewer non-CpG cytosines and thus selecting against incomplete conversion less efficiently. For this reason, we recommend designing primers containing many non-CpG cytosines and preferably right at the 3’ end for at least one of the two primers as is the case for our *CDH1* primers.

If primers select strongly against incomplete conversion by the inclusion of multiple Ts derived from non-CpG Cs (as our *CDH1* primers do) and the detected methylation levels are relatively high, it may be of interest to have CpG positions in between the primers, since this allows assessment of whether the methylation between the primers is heterogeneous or homogeneous by HRM analysis. If a complex melting pattern extending to the left is observed this is an indication that the CpG sites in between the primers are not consistently methylated. In our *CDH1* SMART-MSP assay, we have shown that this can be assessed by running the assay at different annealing temperatures. If methylation levels are estimated to be lower when using higher annealing temperatures, this indicates that some of the CpG sites under the primers are not co-methylated. We interpret our *CDH1* results as indicating that the CpG site close to the 5’ end of the forward primer does not need to be co-methylated when using 65°C annealing temperature. However, at 68°C, methylation levels were estimated to be slightly lower, indicating that this site now needs to be methylated for amplification to occur. Thus, when heterogeneously methylated DNA is analysed by quantitative MSP based methods, the estimated methylation levels will be dependent on the annealing temperature and the number and positions of the CpG sites in the primers. For very accurate quantification, methods capable of reading the methylation status of individual epialleles, such as digital MS-HRM and sequencing [29], digital MethyLight [30], clonal bisulfite genomic sequencing [31], and next generation sequencing of bisulfite modified DNA [32] will be necessary [33].

Interestingly, the observed individual variation of methylation levels for the studied healthy individuals, indicates that it may be of interest to further study the concept that individual variation in methylation propensity can be found in normal peripheral blood mononuclear cells. The results indicate that some individuals may have a significant burden of epimutations as they have increased methylation over a large number of methylatable promoter regions. In particular, samples 10 and 11 showed higher levels of methylation than the rest of the samples and had the most genes methylated as well. By contrast, samples 26, 27, 35, 37, 39, 42, 46, 47 and 48 showed low methylation levels and the methylation was restricted to only a few genes. The reasons for this remain unknown. Importantly, our results eliminate the trivial interpretation that the apparent hypermethylator phenotype is an artifact due to incomplete bisulfite conversion. Defects in methyl metabolism remain a possibility [34].

The varying levels of background methylation may reflect cancer predisposition. Each methylated allele represents an epimutation and if such epimutations affect genes whose inactivation predisposes to cancer development (as is the case for this gene panel), and these epimutations also occur in the target tissues for those cancers, the risk of developing cancer will be increased [34]. Prospective studies will be needed to determine if elevated methylation levels in fact predispose to cancer, and a larger sample cohort will be needed to address these questions appropriately. Interestingly, several studies have recently shown that DNA from peripheral blood may be differentially methylated between cancer patients and normal controls [35-37].

In conclusion, because DNA from normal leukocytes contributes to the templates prepared from body fluids that can be non-invasively used for PCR based analysis [6, 38], biomarkers methylated in normal blood may be less specific, especially when using non-quantitative methods. Thus, methylation levels should be assessed quantitatively for discrimination of low-level methylation in healthy cells from methylated DNA derived from cancer cells.

## MATERIALS AND METHODS

### Samples

The investigations were performed after approval by the Peter MacCallum Cancer Centre Ethics of Human Research committee (Projects 02/70 and 02/26). Peripheral blood samples from blood donors were obtained after informed consent from the Australian Red Cross Blood Service.

### DNA extraction and bisulphite modification

Mononuclear cells from 3-5 mL of peripheral blood were prepared using Lymphoprep (Nyegaard, Oslo, Norway) and DNA was extracted using a salting out method [39]. Universal Methylated DNA (Chemicon, Millipore, Billerica, MA) was used as a fully methylated control. Whole genome amplified DNA from peripheral blood cells of normal individuals was used as unmethylated DNA as described previously [12]. Standard dilution series of 100%, 10%, 1%, 0.1%, 0.05% and 0% methylation levels were prepared by diluting the fully methylated DNA into unmethylated DNA. DNA was quantified with a NanoDrop ND-1000 spectrophotometer (NanoDrop Technologies, Wilmington, DE). 500 ng of genomic DNA or WGA product was subjected to bisulphite conversion with the EpiTect^®^ 96 Bisulphite kit (Qiagen, Hilden, Germany) according to the manufacturer’s instructions.

### SMART-MSP primer design

The amplicons were designed to allow the HRM analysis to assess the conversion status of non-CpG cytosines between the primers [12]. The primers overlaid a minimum of 2 CpG sites with one of the cytosines of a CpG site placed at or adjacent to the 3’ end. Non-CpG cytosines were included in the primer sequences to select against incompletely converted sequences, and at least one of these was placed as close to the 3’ end as possible. The primer sequences, genomic regions spanned, amplicon sizes and the annealing temperatures are found in Table 1. The primers for *DAPK1* and *CDKN2A* (p16^INK4a^) were previously published [12].

### PCR and HRM conditions for the SMART-MSP assays

PCR cycling and HRM analysis were performed on the Rotor-Gene 6000™ (Corbett Research, Sydney, Australia). SYTO^®^ 9 was used as the intercalating dye (Life Technologies, Carlsbad, CA). The reaction mixtures consisted of 25 ng of bisulphite modified template (pre bisulfite conversion amount), 1x PCR buffer, 2.5 mmol/L MgCl_2_ final (3 mmol/L in the *CDH1* assay), 200 nmol/L of each primer, 200 μmol/L of each dNTP, 5 μmol/L of SYTO 9, 0.5U of HotStarTaq (Qiagen) in a final volume of 20 μL.

The PCR comprised one cycle of 95°C for 15 min, followed by 45 cycles of 95°C for 20 s, annealing at the appropriate temperature (Table 1) for 30 s, 72°C for 30 s, and one cycle of 95°C for 1 min. HRM was performed from 60°C to 90°C, with a temperature increase at the rate of 0.2°C per second for all assays. The annealing temperature was experimentally determined for each assay to ensure only methylated templates were amplified. For each assay, a standard dilution series was run to assess the quantitative accuracy and sensitivity. Fully methylated and fully unmethylated control (WGA product), unmodified control, and no template control were also included in every run. All samples were analysed in duplicate.

### The CDH1 MethyLight assay

The same forward primer was used for the MethyLight assay as for the SMART-MSP assay. The reverse primer sequence was: 5’-cgctaattaactaaaaattcacctaccg-3’. The probe sequence was FAM-5’-ttcgcgttgttgattgg-3’-BHQ (IDT). The two CpG sites between the primers are covered by the probe. The reaction mixtures consisted of 25 ng of bisulphite modified DNA, 1x PCR buffer, 250 nmol/L of probe, 3 mmol/L MgCl_2_, 200 nmol/L of each primer, 200 μmol/L of each dNTP, and 0.5U of HotStarTaq (Qiagen) (5U/μL) in a total volume of 20 μL. The PCR comprised one cycle of 95°C for 15 min, 45 cycles of 95°C for 20 s and 64°C for 40 s. PCR was performed on the Rotor-Gene 6000. All samples were analysed in duplicate. In some reactions the probe was omitted and the primers were used for SMART-MSP to allow assessment of the methylation status of the two CpG sites in the region of the probe by HRM analysis.

### Real-Time PCR quantification

The *COL2A1* control assay amplifying a CpG free region was used to normalise for DNA input after bisulphite conversion in the real-time PCR quantification [12]. The relative 2^(-delta delta CT)^ quantification approach [40] was used. The C_T_ value of the control *COL2A1* assay (Table 1) is subtracted from the C_T_ value of the target gene for the calibrator sample (the 100% methylated standard). For each sample, this value is then subtracted from the value resulting from the C_T_ value of the target gene minus the C_T_ value for the *COL2A1* control assay. For this approach to be valid, the amplification efficiencies of the target and the control must be approximately equal [40]. The take-off values (defined as the cycle at which the second derivative is at 20% of the maximum level) given by comparative quantification (using the Rotor-Gene 6000 Series Software, version 1.7.61) were used as C_T_ values in the calculations.

### Sequencing

Sequencing was used to verify a higher melting temperature was due to incomplete conversion of some of the non-CpG cytosines in between the *APC* SMART-MSP primers. For this purpose, a second amplification was performed with m13 tagged *APC* SMART-MSP primers. These products were sequenced using BigDye terminator chemistry v3.1 on an ABI 3730 (Applied Biosystems).

## References

[1] Hochedlinger K, Blelloch R, Brennan C, Yamada Y, Kim M, Chin L, Jaenisch R (2004). Reprogramming of a melanoma genome by nuclear transplantation. Genes & Development.

[2] Schuebel KE, Chen W, Cope L, Glockner SC, Suzuki H, Yi JM, Chan TA, Van Neste L, Van Criekinge W, van den Bosch S, van Engeland M, Ting AH, Jair K, Yu W, Toyota M, Imai K (2007). Comparing the DNA hypermethylome with gene mutations in human colorectal cancer. PLoS Genetics.

[3] Ushijima T, Asada K (2010). Aberrant DNA methylation in contrast with mutations. Cancer Science.

[4] Wade PA (2001). Methyl CpG binding proteins: coupling chromatin architecture to gene regulation. Oncogene.

[5] Laird PW (2003). The power and the promise of DNA methylation markers. Nature Reviews Cancer.

[6] Shi H, Wang MX, Caldwell CW (2007). CpG islands: their potential as biomarkers for cancer. Expert Review of Molecular Diagnostics.

[7] Cottrell SE, Laird PW (2003). Sensitive detection of DNA methylation. Annals of the New York Academy of Sciences.

[8] Mikeska T, Bock C, Do H, Dobrovic A DNA methylation biomarkers in cancer: progress towards clinical implementation. Expert Review of Molecular Diagnostics (accepted for publication).

[9] Dobrovic A, Coleman WB, Tsongalis GJ (2005). Methods for Analysis of DNA Methylation. Molecular diagnostics for the clinical laboratorian.

[10] Kristensen LS, Hansen LL (2009). PCR-based methods for detecting single-locus DNA methylation biomarkers in cancer diagnostics, prognostics, and response to treatment. Clinical Chemistry.

[11] Herman JG, Graff JR, Myohanen S, Nelkin BD, Baylin SB (1996). Methylation-specific PCR: a novel PCR assay for methylation status of CpG islands. Proceedings of the National Academy of Sciences of the United States of America.

[12] Kristensen LS, Mikeska T, Krypuy M, Dobrovic A (2008). Sensitive Melting Analysis after Real Time- Methylation Specific PCR (SMART-MSP): high-throughput and probe-free quantitative DNA methylation detection. Nucleic Acids Research.

[13] Rand K, Qu W, Ho T, Clark SJ, Molloy P (2002). Conversion-specific detection of DNA methylation using real-time polymerase chain reaction (ConLight-MSP) to avoid false positives. Methods.

[14] Shaw RJ, Akufo-Tetteh EK, Risk JM, Field JK, Liloglou T (2006). Methylation enrichment pyrosequencing: combining the specificity of MSP with validation by pyrosequencing. Nucleic Acids Research.

[15] Eads CA, Danenberg KD, Kawakami K, Saltz LB, Blake C, Shibata D, Danenberg PV, Laird PW (2000). MethyLight: a high-throughput assay to measure DNA methylation. Nucleic Acids Research.

[16] Kristensen LS, Wojdacz TK, Thestrup BB, Wiuf C, Hager H, Hansen LL (2009). Quality assessment of DNA derived from up to 30 years old formalin fixed paraffin embedded (FFPE) tissue for PCR-based methylation analysis using SMART-MSP and MS-HRM. BMC Cancer.

[17] Wittwer CT, Reed GH, Gundry CN, Vandersteen JG, Pryor RJ (2003). High-resolution genotyping by amplicon melting analysis using LC Green. Clinical Chemistry.

[18] Gudnason H, Dufva M, Bang DD, Wolff A (2007). Comparison of multiple DNA dyes for real-time PCR: effects of dye concentration and sequence composition on DNA amplification and melting temperature. Nucleic Acids Research.

[19] Belinsky SA, Nikula KJ, Palmisano WA, Michels R, Saccomanno G, Gabrielson E, Baylin SB, Herman JG (1998). Aberrant methylation of p16(INK4a) is an early event in lung cancer and a potential biomarker for early diagnosis. Proceedings of the National Academy of Sciences of the United States of America.

[20] Palmisano WA, Divine KK, Saccomanno G, Gilliland FD, Baylin SB, Herman JG, Belinsky SA (2000). Predicting lung cancer by detecting aberrant promoter methylation in sputum. Cancer Research.

[21] Hoque MO, Topaloglu O, Begum S, Henrique R, Rosenbaum E, Van Criekinge W, Westra WH, Sidransky D (2005). Quantitative methylation-specific polymerase chain reaction gene patterns in urine sediment distinguish prostate cancer patients from control subjects. Journal of Clinical Oncology.

[22] Wang YC, Yu ZH, Liu C, Xu LZ, Yu W, Lu J, Zhu RM, Li GL, Xia XY, Wei XW, Ji HZ, Lu H, Gao Y, Gao WM, Chen LB (2008). Detection of RASSF1A promoter hypermethylation in serum from gastric and colorectal adenocarcinoma patients. World Journal of Gastroenterol.

[23] Wong EM, Southey MC, Fox SB, Brown MA, Dowty JG, Jenkins MA, Giles GG, Hopper JL, Dobrovic A (2011). Constitutional methylation of the BRCA1 promoter is specifically associated with BRCA1 mutation-associated pathology in early-onset breast cancer. Cancer Prev Res (Phila).

[24] Evron E, Dooley WC, Umbricht CB, Rosenthal D, Sacchi N, Gabrielson E, Soito AB, Hung DT, Ljung B, Davidson NE, Sukumar S (2001). Detection of breast cancer cells in ductal lavage fluid by methylation-specific PCR. Lancet.

[25] Lombaerts M, Middeldorp JW, van der Weide E, Philippo K, van Wezel T, Smit VT, Cornelisse CJ, Cleton-Jansen AM (2004). Infiltrating leukocytes confound the detection of E-cadherin promoter methylation in tumors. Biochemical and Biophysical Research Communications.

[26] Reddy AN, Jiang WW, Kim M, Benoit N, Taylor R, Clinger J, Sidransky D, Califano JA (2003). Death-associated protein kinase promoter hypermethylation in normal human lymphocytes. Cancer Research.

[27] Liew M, Pryor R, Palais R, Meadows C, Erali M, Lyon E, Wittwer C (2004). Genotyping of single-nucleotide polymorphisms by high-resolution melting of small amplicons. Clinical Chemistry.

[28] Kristensen LS, Dobrovic A (2008). Direct genotyping of single nucleotide polymorphisms in methyl metabolism genes using probe-free high-resolution melting analysis. Cancer Epidemiology, Biomarkers & Prevention.

[29] Candiloro IL, Mikeska T, Hokland P, Dobrovic A (2008). Rapid analysis of heterogeneously methylated DNA using digital methylation-sensitive high resolution melting: application to the CDKN2B (p15) gene. Epigenetics Chromatin.

[30] Weisenberger DJ, Trinh BN, Campan M, Sharma S, Long TI, Ananthnarayan S, Liang G, Esteva FJ, Hortobagyi GN, McCormick F, Jones PA, Laird PW (2008). DNA methylation analysis by digital bisulfite genomic sequencing and digital MethyLight. Nucleic Acids Research.

[31] Frommer M, McDonald LE, Millar DS, Collis CM, Watt F, Grigg GW, Molloy PL, Paul CL (1992). A genomic sequencing protocol that yields a positive display of 5-methylcytosine residues in individual DNA strands. Proceedings of the National Academy of Sciences of the United States of America.

[32] Taylor KH, Kramer RS, Davis JW, Guo J, Duff DJ, Xu D, Caldwell CW, Shi H (2007). Ultradeep bisulfite sequencing analysis of DNA methylation patterns in multiple gene promoters by 454 sequencing. Cancer Research.

[33] Mikeska T, Candiloro IL, Dobrovic A (2010). The implications of heterogeneous DNA methylation for the accurate quantification of methylation. Epigenomics.

[34] Dobrovic A, Kristensen LS (2009). DNA methylation, epimutations and cancer predisposition. The International Journal of Biochemistry & Cell Biology.

[35] Pedersen KS, Bamlet WR, Oberg AL, de Andrade M, Matsumoto ME, Tang H, Thibodeau SN, Petersen GM, Wang L (2011). Leukocyte DNA methylation signature differentiates pancreatic cancer patients from healthy controls. PLoS One.

[36] Wang L, Aakre JA, Jiang R, Marks RS, Wu Y, Chen J, Thibodeau SN, Pankratz VS, Yang P (2010). Methylation markers for small cell lung cancer in peripheral blood leukocyte DNA. Journal of Thoracic Oncology.

[37] Teschendorff AE, Menon U, Gentry-Maharaj A, Ramus SJ, Gayther SA, Apostolidou S, Jones A, Lechner M, Beck S, Jacobs IJ, Widschwendter M (2009). An epigenetic signature in peripheral blood predicts active ovarian cancer. PLoS One.

[38] Bhatia K, Siraj AK, Hussain A, Bu R, Gutierrez MI (2003). The tumor suppressor gene 14-3-3 sigma is commonly methylated in normal and malignant lymphoid cells. Cancer Epidemiology, Biomarkers & Prevention.

[39] Miller SA, Dykes DD, Polesky HF (1988). A simple salting out procedure for extracting DNA from human nucleated cells. Nucleic Acids Research.

[40] Livak KJ, Schmittgen TD (2001). Analysis of relative gene expression data using real-time quantitative PCR and the 2(-Delta Delta C(T)) method. Methods.

